# Enhanced Antitumor Immune Response in 2′-5′ Oligoadenylate Synthetase-Like 1- (OASL1-) Deficient Mice upon Cisplatin Chemotherapy and Radiotherapy

**DOI:** 10.1155/2019/7596786

**Published:** 2019-03-31

**Authors:** Chan Kyu Sim, Jung Hoon Lee, In-Jeoung Baek, Sang-Wook Lee, Myeong Sup Lee

**Affiliations:** ^1^Laboratory of Molecular Immunology and Medicine, Department of Biomedical Sciences, University of Ulsan College of Medicine, Asan Medical Center, Seoul 05505, Republic of Korea; ^2^Department of Convergence Medicine, Asan Institutes for Life Sciences, Asan Medical Center, University of Ulsan College of Medicine, Seoul 05505, Republic of Korea; ^3^Department of Radiation Oncology, University of Ulsan College of Medicine, Asan Medical Center, Seoul 05505, Republic of Korea

## Abstract

Type I interferon (IFN-I) plays a critical role in the antitumor immune response. In our previous study, we showed that IFN-I-inducible 2′-5′ oligoadenylate synthetase-like 1 (OASL1) negatively regulated IFN-I production upon tumor challenge similar to that of viral infection. Thus, OASL1-deficient (*Oasl1* ^−*/*−^) mice were more resistant to implanted tumor growth than wild-type (WT) mice. In this study, we investigated whether targeting or suppressing OASL1 could show synergistic effects on tumor clearance with conventional cancer therapies (such as chemotherapy and radiotherapy) using *Oasl1* ^−*/*−^ mice and a transplantable lung metastatic tumor cell model. Upon treatment with the anticancer drug cisplatin, we found that *Oasl1* ^−*/*−^ mice showed enhanced resistance to injected tumors compared to untreated *Oasl1* ^−*/*−^ mice. Similarly, irradiated *Oasl1* ^−*/*−^ mice showed better resistance to tumor challenge than untreated *Oasl1* ^−*/*−^ mice. Additionally, we found that *Oasl1* ^−*/*−^ mice applied with both types of the cancer therapies contained more cytotoxic effector cells, such as CD8^+^ T cells and NK cells, and produced more cytotoxic effector cytokine IFN-*γ* as well as IFN-I in their tumor-containing lungs compared to untreated *Oasl1* ^−*/*−^ mice. Collectively, these results show that targeting OASL1 together with conventional cancer therapies could be an effective strategy to enhance treatment efficacy.

## 1. Introduction

Conventional cancer therapies, such as chemotherapy and radiotherapy, are still the main treatment options in the clinic [1]. More recently, cancer immunotherapy, which boosts the host's immune response against tumor cells, has emerged as an additional treatment alternative [2]. Popular cancer immunotherapeutic approaches include administration of monoclonal antibodies (Abs) targeting immune checkpoints, adoptive transfer of engineered T cells or tumor-infiltrating lymphocytes (TILs), tumor vaccination, and injection of immune-boosting cytokines or immune adjuvants [3, 4]. Since a single cancer treatment is not effective in many cases, combinational therapy using different approaches is becoming an important anticancer strategy to completely eradicate cancers in patients [5, 6]. Therefore, diverse combinatorial approaches have been performed in preclinical and clinical investigations [7, 8].

Type I interferon (IFN-I) and its signaling, which are originally known to be essential for antiviral responses, have also been demonstrated to be critical for an effective antitumor immune response [9–11]. Thus, deliveries of exogenous IFN-I and IFN-I-inducing pathogen-associated molecular patterns (PAMPs) were shown to induce enhanced antitumor immune responses [12–16]. Since negative regulators have been shown to affect IFN-I production during viral infections, inhibition of these negative regulators could increase the antitumor immune response [17, 18]. Previously, we have shown that IFN-I-inducible 2′-5′ oligoadenylate synthetase-like 1 (OASL1), a specific inhibitor of IRF7 (master transcription factor (TF) for IFN-I) translation, negatively regulates IFN-I production upon tumor challenge, similar to those of viral infections [19–22]. As a result, OASL1-deficient (*Oasl1* ^−*/*−^) mice were more resistant to implanted tumor growth than wild-type (WT) mice [22].

In this study, we evaluated whether standard cancer therapies, such as chemotherapy and radiotherapy, would enhance resistance to implanted tumors in *Oasl1* ^−/−^ mice. We found that *Oasl1* ^−/−^ mice showed more resistance to implanted tumors when treated with cisplatin chemotherapy and radiotherapy than untreated *Oasl1* ^−/−^ mice. This result indicates that OASL1 inhibition can be well integrated with other cancer therapies as a combinatorial treatment.

## 2. Materials and Methods

### 2.1. Mice and Cells

*Oasl1* ^−*/*−^ mice [19] backcrossed to C57BL/6 (The Jackson Laboratory) for ten generations and their littermates were housed in a specific pathogen-free facility. Animal studies were approved by the Institutional Animal Care and Use Committee of Asan Institute for Life Sciences (permit number: 2018-14-046). TC-1, a syngeneic lung epithelial tumor cell line (a gift from T.C. Wu, Johns Hopkins University) established from C57BL/6 lung epithelial cells by expressing HPV oncogenic protein E7 [23], was used. Prior to injection, the cell line was cultured in DMEM supplemented with 10% FBS at 37°C under 5% CO_2_.

### 2.2. *In Vivo* Tumor Model, Therapy, and Tumor Burden Analysis

To establish an *in vivo* tumor model, TC-1 tumor cells (10^6^ cells per mouse) were injected intravenously into the tail of six-to-ten-week-old littermate C57BL/6 wild-type (WT) and *Oasl1* ^−*/*−^ mice, and mouse health and weight were monitored daily before analysis. A single chemotherapy treatment was applied to mice by intraperitoneal (i.p.) injection of the drug cisplatin (P4394, Sigma-Aldrich) 6 days post-TC-1 tumor injection (d.p.i.). A single radiotherapy treatment was applied to mice at 7 d.p.i. through whole body irradiation using an X-ray irradiator (X-RAD 320, Precision X-Ray Inc.) following anesthesia with Avertin (a mixture of 2,2,2-tribromoethanol (T48402, Sigma-Aldrich) and *tert*-amyl alcohol (PHR1667, Sigma-Aldrich)). To analyze the tumor burden, the left lobe of the lungs was soaked in Bouin's solution (HT10132, Sigma-Aldrich) for more than one day and weighed. Lungs with tumor nodules are heavier than normal lungs.

### 2.3. FACS Analysis on Immune Cells in Tumor-Containing Lungs

For FACS analysis, the lungs (the right inferior lobe of the lung containing tumor nodules) were collected at the indicated days after TC-1 injection. Subsequently, the lungs were chopped and digested as previously described using 1 mg/mL type II collagenase (C6885, Sigma-Aldrich) and 1 U/mL DNase I (04536282001, Roche) to obtain single cells [22]. To remove red blood cells, the digested single cells were incubated with ACK lysing buffer (1 mL/tissue, A10492-01, Life Technologies) for 5 min at room temperature and then washed twice with cold DPBS. For staining, the prepared single cells were first Fc blocked (0.5 *μ*L/10^6^ cells) in FACS buffer (DPBS containing 2% FBS) for 20 min at 4°C with CD16/CD32 antibodies (Abs) (2.4G2, BD Pharmingen). Dead cell staining with the Aqua fluorescent reactive dye from the Live/Dead Fixable Dead Cell Stain Kit (L34960, Invitrogen) and surface staining were performed for 30 min at 4°C in the FACS buffer. If necessary, intracellular staining was next performed for 20 min at 4°C using a BD Cytofix/Cytoperm solution kit (554714, BD Pharmingen). The surface staining Abs from BD Pharmingen were CD3e (145-2C11), CD4 (RM4-5), CD8a (53-6.7), CD19 (1D3), NK1.1 (PK136), CD45 (30-F11), CD11c (HL-3), CD45R/B220 (RA3-6B2), and Gr-1 (RB6-8C5); the Abs from eBioscience were CD11b (M1/70) and F4/80 (BM8); and the Ab from Miltenyi Biotec was PDCA-1 (JF05-1C2.4.1). Intracellular staining Abs Foxp3 (FJK-16S) and CD68 (FA-11) were from eBioscience. Stained samples were assessed using FACSCanto II (BD Biosciences) and further analyzed using FlowJo software (Tree Star).

### 2.4. FACS Analysis of Apoptotic Cells in Tumor-Containing Lungs

To detect apoptotic cells within nonhematopoietic cells (CD45^−^) which contain mainly tumor cells, the prepared single cells from tumor-containing lungs were Fc blocked and surface stained with the anti-CD45 Ab as described above. Subsequently, the cells were stained with FITC-conjugated Annexin V and 7-AAD using the FITC Annexin V Apoptosis Detection Kit with 7-AAD (640922, BioLegend) following the manufacturer's recommendation. The stained cells were directly analyzed in the FACSCanto II (BD Biosciences).

### 2.5. RNA Analysis by Quantitative Reverse Transcription PCR (qRT-PCR)

Total RNAs (1.5 *μ*g) purified from tissues using QIAzol RNA isolation reagents (79306, Qiagen) were reverse transcribed for 2 h using oligo dT (20 mer) with SuperScript II Reverse Transcriptase (18064-014, Invitrogen) into cDNAs. Quantitative PCR was performed in the CFX Connect Real-Time PCR Detection System (Br185-5200, Bio-Rad) using gene-specific forward (F) and reverse (R) primers by detecting PCR products with SYBR® Green I gel stain dye (S-7567, Life Technologies) as previously described [22, 24]. The following primers were used: *Gapdh* F: GGCAAATTCAACGGCACAGTCAAG and R: TCGCTCCTGGAAGATGGTGATGG; *IFNg* F: GGCCATCAGCAACAACATAAGCGT and R: TGGGTTGTTGACCTCAAACTTGGC; *IFNb1* F: CCACTTGAAGAGCTATTACTG and R: AATGATGAGAAAGTTCCTGAAG; *IFNa5* F: AGGACTCATCTGCTGCATGGAATG and R: CACACAGGCTTTGAGGTCATTGAG; *CXCL9* F: ACATCAGGCTAGGAGTGGTG and R: CACAAGGCTCACGCACAC; *CXCL10* F: CATGAACCCAAGTGCTGCCGTCA and R: TGGATGCAGTTGCAGCGGACCGT; *CXCL11* F: ATCTGGGCCACAGCTGCTCAAG and R: CTCGATCTCTGCCATTTTGACGGCTT; and *XCL1* F: GAAGAGAGTAGCTGTGTGAACTTACAAAC and R: CCCATTTGGCTTCTGGATCAGCACA. The mRNA expression level of each gene was normalized to *Gapdh*.

### 2.6. Statistical Analysis

All data are presented as mean ± standard deviation. All statistical analyses were performed using a two-tailed unpaired Student *t* test. *p* < 0.05 was considered statistically significant.

## 3. Results

### 3.1. Cisplatin-Treated *Oasl1* ^−*/*−^ Mice Are More Resistant to TC-1 Lung Metastatic Tumor Challenge

**Figure 1:**
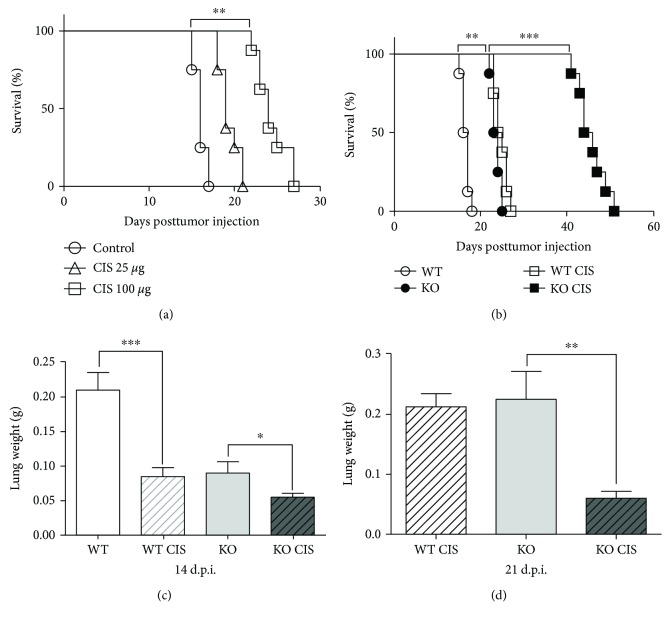
Cisplatin-treated *Oasl1^−/−^* mice are more resistant to TC-1 lung metastatic tumor challenge. Wild-type (WT) or *Oasl1^−/−^* (KO) mice were intravenously injected with TC-1 cells (10^6^ cells per mouse), and at 6 days posttumor cell injection (d.p.i.), cisplatin (CIS) was intraperitoneally injected or not. (a) Survival of cisplatin-treated (CIS 25 *μ*g or CIS 100 *μ*g/mice) or untreated (control) TC-1 tumor-bearing WT mice (*n* = 8 per group) was monitored until the indicated day posttumor cell injection. (b) Survival of cisplatin-treated (CIS 100 *μ*g/mice) or untreated TC-1 tumor-bearing WT (WT) and *Oasl1^−/−^* mice (KO) (*n* = 8 per group) was observed until the indicated day posttumor cell injection. (c, d) The weights of lungs from cisplatin-treated (CIS 100 *μ*g/mice) or untreated, TC-1 tumor-bearing WT and *Oasl1^−/−^* mice (*n* = 4 per group) were measured at 14 (c) and 21 d.p.i. (d). ^∗^ *p* < 0.05, ^∗∗^ *p* < 0.01, and ^∗∗∗^ *p* < 0.001. Data are representative of at least three independent experiments.

To explore whether anticancer chemotherapy can further augment the resistance of *Oasl1* ^−*/*−^ mice to tumor challenge, we employed the TC-1 lung metastasis tumor model used in our previous study, in which TC-1 tumor cells that were introduced into the systemic circulation were deposited in the lung to grow and kill the mice [22, 23]. We chose an anticancer chemotherapeutic agent, cisplatin, one of the most widely used chemotherapeutic agent in clinical practice, for the study [25]. We first determined a therapeutically effective dose of cisplatin in our TC-1 tumor model by a single intraperitoneal (i.p.) injection of cisplatin in wild-type (WT) mice at 6 days post-TC-1 tumor cell injection (d.p.i.) and monitoring survival. Briefly, 25 *μ*g cisplatin-treated WT mice showed a mild improvement in survival (several days), while 100 *μ*g cisplatin-treated WT mice showed a strong survival improvement (about 8 days) (Figure 1(a)). Thus, we chose 100 *μ*g for all of the following experiments. Subsequently, we treated TC-1-injected *Oasl1* ^−*/*−^ mice and WT mice with cisplatin once at 6 d.p.i. and monitored for survival. As previously shown [22], TC-1-injected *Oasl1* ^−*/*−^ mice survived about a week longer than WT mice (Figure 1(b)). When cisplatin was treated, *Oasl1* ^−*/*−^ mice survived much longer (more than 2 weeks) than untreated *Oasl1* ^−*/*−^ mice (Figure 1(b)).

To determine whether the survival difference was caused by a difference in tumor burden, lung weights, which increase with tumor cell load, were analyzed in mice at 14 d.p.i. (when all 4 groups of mice were still alive) and 21 d.p.i. (when cisplatin-untreated *Oasl1* ^−*/*−^ mice were still alive). As previously reported, at 14 d.p.i., lung weights of WT mice were heavier than *Oasl1* ^−*/*−^ mice. As expected, lung weights of cisplatin-treated WT and cisplatin-treated *Oasl1* ^−*/*−^ mice were lighter than untreated WT and *Oasl1* ^−*/*−^ mice, respectively (Figure 1(c)). Consistently, at 21 d.p.i., cisplatin-treated *Oasl1* ^−*/*−^ mice showed much lighter lungs than untreated *Oasl1* ^−*/*−^ mice (Figure 1(d)). These results together indicate that cisplatin-treated *Oasl1* ^−*/*−^ mice demonstrate a better antitumor response to metastatic tumor implantation than untreated *Oasl1* ^−*/*−^ mice.

### 3.2. Tumor-Containing Lungs of Cisplatin-Treated *Oasl1* ^−*/*−^ Mice Contain More Cytotoxic Effector Immune Cells

**Figure 2:**
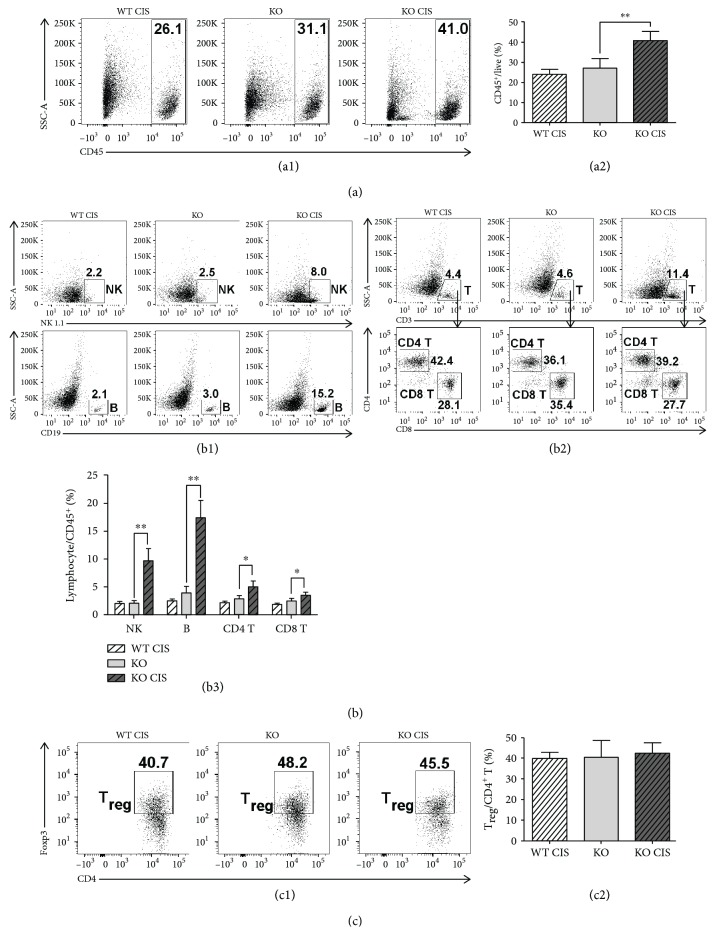
Cisplatin-treated *Oasl1^−/−^* mice contain more major lymphocyte populations in their tumor-containing lungs than untreated *Oasl1^−/−^* mice at 21 d.p.i. WT and *Oasl1^−/−^* (KO) mice were intravenously injected with TC-1 cells (10^6^/mouse). At 6 d.p.i., cisplatin-treatment groups of tumor-bearing WT and *Oasl1^−/−^* mice were intraperitoneally injected with cisplatin (CIS, 100 *μ*g). At 21 d.p.i., the lungs of the mice (*n* = 4 per group) were collected, and the lung-derived single cells were analyzed by FACS. (a) Representative FACS data showing the percentage of CD45^+^ hematopoietic cells among live cells (a1) and summary showing CD45^+^ cell percentage in the tumor-containing lung (a2). (b) Representative FACS data showing the percentages of NK cells (NK1.1^+^) and B cells (CD19^+^) and the percentages of T cells (CD3^+^) among CD45^+^ cells and those of CD4 T cells (CD3^+^CD4^+^) and CD8 T cells (CD3^+^CD8^+^) within the T cells (b1 and b2); summary showing the percentage of lymphocyte subsets among CD45^+^ cells in the lung (b3). (c) Representative FACS data (c1) and summary (c2) showing the percentage of T_reg_ (CD4^+^Foxp3^+^) among CD4^+^ T cells in the lung. ^∗^ *p* < 0.05 and ^∗∗^ *p* < 0.01. Data are representative of at least three independent experiments.

To identify the cause of the enhanced resistance in cisplatin-treated *Oasl1* ^−*/*−^ mice, the major immune cell composition of tumor-containing lungs was analyzed at 21 d.p.i. (when cisplatin-untreated *Oasl1* ^−*/*−^ mice were still alive) by FACS. The percentage of CD45^+^ hematopoietic cells in the lungs of cisplatin-treated *Oasl1* ^−*/*−^ mice at 21 d.p.i. was higher (about 1.5-fold) than those of untreated *Oasl1* ^−*/*−^ mice (Figures 2(a1) and 2(a2)). Additionally, the proportion of major lymphocytes, such as NK cells, B cells, and CD4^+^ and CD8^+^ T cells, within hematopoietic cells was much higher in the lungs of cisplatin-treated *Oasl1* ^−*/*−^ mice (Figures 2(b1), 2(b2), and 2(b3); see Supplementary Figure 1 for gating strategy). However, the proportion of regulatory T cells (T_reg_; CD3^+^CD4^+^Foxp3^+^) among CD4^+^ T cells was not significantly changed in the lungs of cisplatin-treated *Oasl1* ^−*/*−^ mice compared to that of untreated *Oasl1* ^−*/*−^ mice (Figures 2(c1) and 2(c2)).

**Figure 3:**
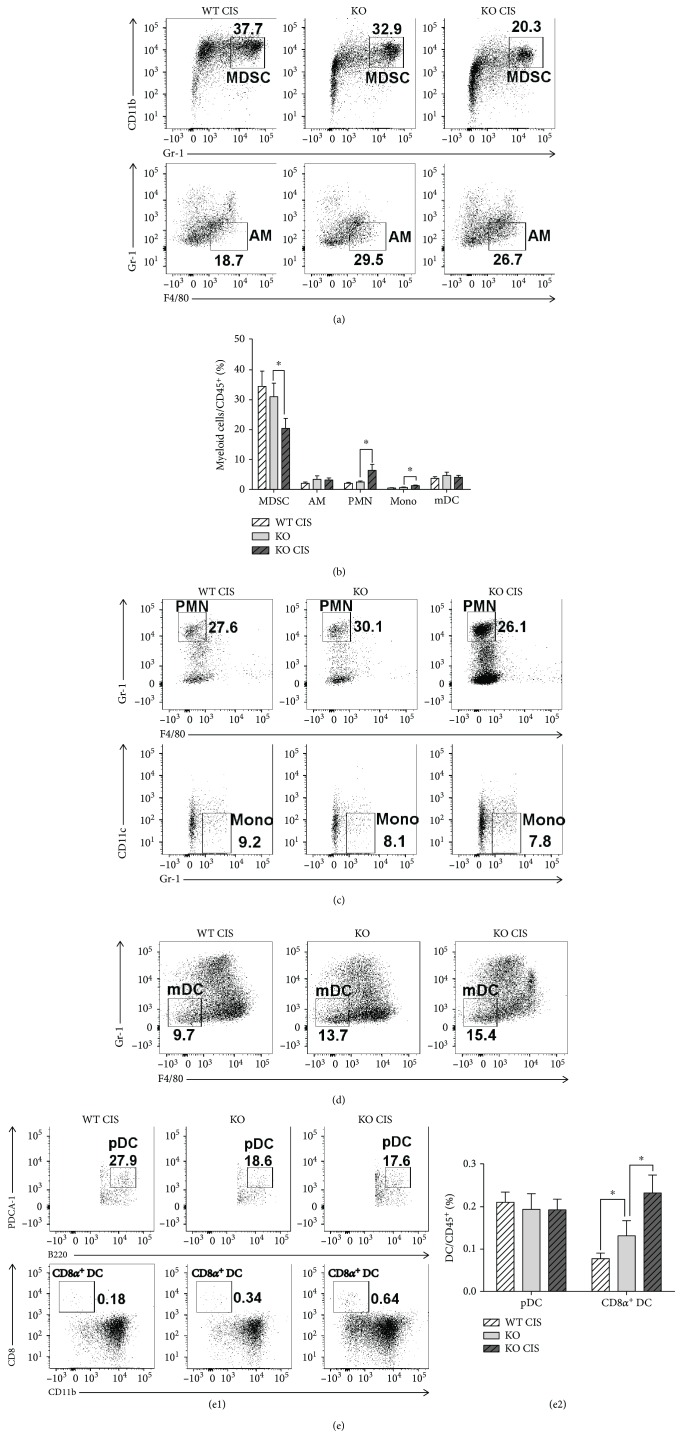
Cisplatin-treated *Oasl1^−/−^* mice contain more CD8*α* ^+^ DC in their tumor-containing lung than untreated-*Oasl1^−/−^* mice. WT and *Oasl1^−/−^* (KO) mice (*n* = 4 per group) were intravenously injected with TC-1 cells (10^6^/mouse). At 6 d.p.i., cisplatin-treatment groups of tumor-bearing WT and *Oasl1^−/−^* mice were intraperitoneally injected with cisplatin (CIS, 100 *μ*g). Subsequently, at 21 d.p.i., lung samples for cisplatin-treated WT mice (WT CIS), untreated *Oasl1^−/−^* mice (KO), and cisplatin-treated *Oasl1^−/−^* mice (KO CIS) were collected, and the tissue-derived single cells were analyzed by FACS. (a–c) Representative FACS data showing the percentage of myeloid subsets, including (a) MDSC and AM and (c) PMN and monocyte (Mono) among parent populations. (b) The summary data showing the percentage of myeloid subsets and mDC within CD45^+^ cells. (d) Representative FACS data showing the percentage of mDC within parent population. (e) Representative FACS data showing the percentage of pDC and CD8*α* ^+^ DC among their parent populations (e1) and summary data showing the percentage of pDC and CD8*α* ^+^ DC within CD45^+^ cells (e2). ^∗^ *p* < 0.05. Data are representative of at least three independent experiments.

Major pulmonary myeloid cell populations were then analyzed at 21 d.p.i. by FACS (see Supplementary Figure 2 for gating strategy) [26]. The proportion of myeloid-derived suppressor cells (MDSCs; broadly defined as CD45^+^Gr-1^+^CD11b^+^ and the most dominant myeloid cells) within CD45^+^ cells in the lungs of cisplatin-treated *Oasl1* ^−*/*−^ mice was lower than untreated *Oasl1* ^−*/*−^ mice (Figures 3(a) and 3(b)). Further, the proportion of alveolar macrophages (AM; CD45^+^CD68^hi^CD11c^+^CD11b^−^F4/80^+^Gr-1^−^) was not significantly different from that of untreated *Oasl1* ^−*/*−^ mice (Figures 3(a) and 3(b)). However, the proportion of polymorphonuclear cells (PMN; CD45^+^CD68^low^CD11b^+^F4/80^−^Gr-1^hi^) and monocytes (Mono; CD45^+^CD68^low^CD11b^+^Gr-1^low^CD11c^−^) was higher than that of untreated *Oasl1* ^−*/*−^ mice (Figures 3(b) and 3(c)). Among the major dendritic cell (DC) populations, the proportions of conventional myeloid DC (mDC; CD45^+^CD68^hi^CD11c^+^F4/80^−^Gr-1^−^) and plasmacytoid DC (pDC; CD45^+^CD11c^+^B220^+^PDCA-1^+^) within CD45^+^ cells in the lungs of cisplatin-treated *Oasl1* ^−*/*−^ mice were not significantly different from those of untreated *Oasl1* ^−*/*−^ mice (Figures 3(b)–3(e1) and 3(e2)). However, the percentage of CD8*α* ^+^ DC (CD45^+^CD11c^+^B220^−^CD8*α* ^+^CD11b^−^) within CD45^+^ cells in the lungs of cisplatin-treated *Oasl1* ^−*/*−^ mice was much higher than that of untreated *Oasl1* ^−*/*−^ mice (Figure 3(e1) and 3(e2)).

The observation that CD8^+^ T and NK cells (cytotoxic effector cells), as well as CD8*α* ^+^ DCs (major cells cross-presenting tumor antigen to CD8^+^ T cells) [27], were present in a much higher proportion, yet the proportion of MDSCs and T_reg_ (immunosuppressive cells) within CD45^+^ cells were lower or unchanged relative to untreated *Oasl1* ^−*/*−^ mice, indicates that more effective tumor antigen cross-presentation and cytotoxic CD8^+^ T cell production occur in the lungs of cisplatin-treated *Oasl1* ^−*/*−^ mice.

### 3.3. Cisplatin-Treated *Oasl1* ^−*/*−^ Mice Produce More Cytotoxic Effector Cytokine IFN-*γ*, IFN-I, and Apoptotic Cells in the Tumor-Containing Lungs

**Figure 4:**
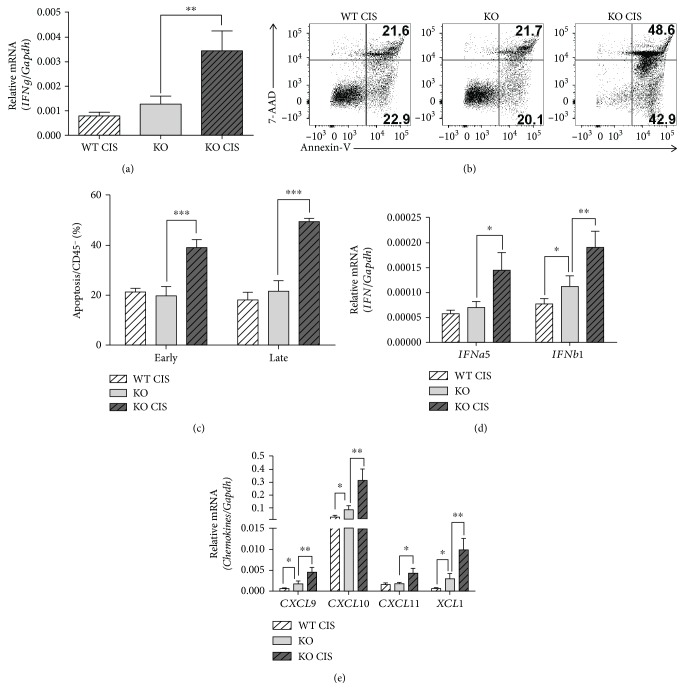
Cisplatin-treated *Oasl1^−/−^* mice have more cytotoxic effector cytokine IFN-*γ*, IFN-I, and apoptotic cells in the tumor-containing lung. WT and *Oasl1^−/−^* mice (KO) were TC-1 injected intravenously (10^6^ per mouse), and at 6 d.p.i., cisplatin-treatment groups of tumor-bearing WT and *Oasl1^−/−^* mice were intraperitoneally injected with cisplatin (CIS, 100 *μ*g). At 21 d.p.i., the right middle lobe of the lung for RNA analysis and right inferior lobe of the lung for apoptosis analysis were collected from cisplatin-treated WT (WT CIS), untreated-*Oasl1^−/−^* (KO), and cisplatin-treated *Oasl1^−/−^* mice (KO CIS). (a) Quantitative RT-PCR analysis of IFN-*γ* (*IFNg*) mRNA expression at 21 d.p.i. mRNA expression level (*n* = 4 per group) normalized to *Gapdh* is shown as relative mRNA. (b, c) Representative FACS data showing the percentage of early apoptotic cells (Annexin V^+^/7-AAD^−^) and late apoptotic cells (Annexin V^+^/7-AAD^+^) among CD45^−^ cells (b) and summary data (*n* = 4 per group) showing the percentage of two types of apoptotic cells among CD45^−^ cells (c). (d, e) Quantitative RT-PCR analysis of mRNA expression for *IFNa5* and *IFNb1* (d) and for *CXCL9*, *CXCL10*, *CXCL11*, and *XCL1*, at 21 d.p.i. (e). mRNA expression level (*n* = 4 per group) normalized to *Gapdh* is shown as relative mRNA. ^∗^ *p* < 0.05, ^∗∗^ *p* < 0.01, and ^∗∗∗^ *p* < 0.01. Data are representative of at least three independent experiments.

To indirectly investigate the functionality of the CD8^+^ T and NK cells, the expression of IFN-*γ* (key cytotoxic effector cytokine) was evaluated in the tumor-containing lungs at 21 d.p.i. by qRT-PCR. The expression level of IFN-*γ* mRNA was much higher (about 2.5-fold) in the lungs of cisplatin-treated *Oasl1* ^−*/*−^ mice compared to untreated *Oasl1* ^−*/*−^ mice (Figure 4(a)), suggesting that cytotoxic CD8^+^ T and NK cells together are functionally more active in the lungs of cisplatin-treated *Oasl1* ^−*/*−^ mice. Therefore, enhanced apoptotic cell death is expected in the lungs of cisplatin-treated *Oasl1* ^−*/*−^ mice. To measure apoptotic cell death directly in the tumor-containing lungs, FACS analysis was performed on single cells derived from the lungs at 21 d.p.i., using 7-AAD/Annexin V to detect apoptotic cells. As expected, lungs of cisplatin-treated *Oasl1* ^−*/*−^ mice contained much more early (Annexin V^+^/7-AAD^−^) and late (Annexin V^+^/7-AAD^+^) apoptotic cells within nonhematopoietic cells (CD45^−^ cells, mainly tumor cells) [28, 29] than untreated *Oasl1* ^−*/*−^ mice (Figures 4(b) and 4(c)). These results indicate that cisplatin-treated *Oasl1* ^−*/*−^ mice have more tumor-attacking CD8^+^ T cells and NK cells that can effectively kill growing tumors in the lungs, leading to improved survival of cisplatin-treated *Oasl1* ^−*/*−^ mice.

We previously showed that tumor-challenged *Oasl1* ^−*/*−^ mice expressed more IFN-I in the tumor-containing lungs compared to WT mice [22]. Therefore, we investigated whether cisplatin-treated *Oasl1* ^−*/*−^ mice had increased IFN-I expression in the tumor-containing lungs at 21 d.p.i. The mRNA expression levels of IFN-Is, such as *IFNa5* and *IFNb1*, in the lungs of cisplatin-treated *Oasl1* ^−*/*−^ mice were higher (approximately 1.5-fold) than untreated *Oasl1* ^−*/*−^ mice (Figure 4(d)).

Since IFN-I is known to induce the expression of lymphocyte-recruiting chemokines such as CXCL9, CXCL10, and CXCL11 [30–32], we measured the mRNA expression level of these chemokines in the tumor-containing lungs at 21 d.p.i. The mRNA expression levels were higher (>2-fold) than those in the untreated *Oasl1* ^−*/*−^ mice (Figure 4(e)). In addition, the mRNA expression level of XCL1 (CD8*α* ^+^ DC-recruiting chemokine) [33] was higher in the lungs of cisplatin-treated *Oasl1* ^−*/*−^ mice than in those of untreated *Oasl1* ^−*/*−^ mice (Figure 4(e)). These results together indicate that the higher number of NK, CD8 T, and CD8*α* ^+^ DC present in the lungs of cisplatin-treated *Oasl1* ^−*/*−^ mice is caused, in part, by increased chemokine production in the lungs of cisplatin-treated *Oasl1* ^−*/*−^ mice compared to untreated *Oasl1* ^−*/*−^ mice.

### 3.4. Radiation-Treated *Oasl1* ^−*/*−^ Mice Are More Resistant to TC-1 Lung Metastatic Tumor Challenge

**Figure 5:**
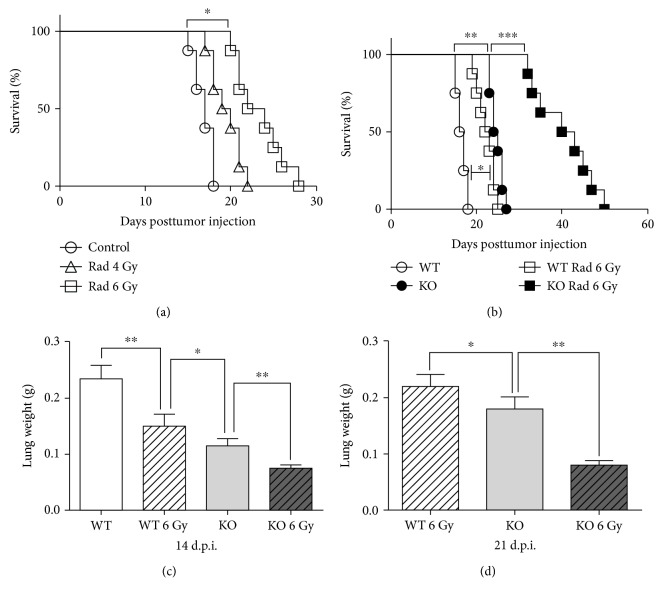
Irradiated *Oasl1^−/−^* mice are more resistant to TC-1 tumor challenge than nonirradiated *Oasl1^−/−^* mice. WT and *Oasl1^−/−^* mice were TC-1 injected intravenously (10^6^ per mouse), and irradiation was performed on day 7 post-TC-1 injection to anesthetized mice. (a) Survival of nonirradiated (control) and irradiated (Rad 4 Gy or Rad 6 Gy) tumor-bearing WT mice was observed until the indicated days to determine the proper irradiation dosage. (b) Survival of nonirradiated (WT and KO) and 6 Gy irradiated tumor-bearing WT (WT Rad 6 Gy) and *Oasl1^−/−^* mice (KO Rad 6 Gy) was monitored until the indicated days. (c, d) The weight of the left lobe of the lung from untreated and 6 Gy irradiated tumor-bearing WT (WT 6 Gy) and *Oasl1^−/−^* mice (KO 6 Gy) was measured at 14 (c) and 21 d.p.i. (d). ^∗^ *p* < 0.05, ^∗∗^ *p* < 0.01, and ^∗∗∗^ *p* < 0.001. Data are representative of at least three independent experiments.

To explore whether radiotherapy, another major conventional cancer therapy, can further augment resistance of *Oasl1* ^−*/*−^ mice to tumor challenge, we first determined the therapeutically effective radiation dosage in our TC-1 lung metastasis model by irradiating WT mice once at 7 d.p.i. and monitoring survival. Briefly, survival of 4 Gy irradiated WT mice was mildly improved (several days) compared to that of untreated WT mice; however, survival of 6 Gy irradiated WT mice was much higher (about a week) (Figure 5(a)). Thus, we chose 6 Gy for further experiments. When we irradiated TC-1-injected *Oasl1* ^−*/*−^ mice at this dosage, the irradiated mice survived longer than nonirradiated *Oasl1* ^−*/*−^ mice (Figure 5(b)). Consistently, tumor burden measured by the lung weights of the mice at 14 d.p.i. was much lighter in the irradiated mice than nonirradiated mice for both *Oasl1* ^−*/*−^ and WT mice (Figure 5(c)). At 21 d.p.i., when nonirradiated *Oasl1* ^−*/*−^ mice were still alive, irradiated *Oasl1* ^−*/*−^ mice also had much lighter lungs than nonirradiated *Oasl1* ^−*/*−^ mice (Figure 5(d)). These results indicate that irradiated *Oasl1* ^−*/*−^ mice demonstrate a better antitumor response to metastatic tumor implantation than untreated *Oasl1* ^−*/*−^ mice.

### 3.5. Radiation-Treated *Oasl1* ^−*/*−^ Mice Contain Higher Cytotoxic Effector Immune Cells in the Tumor-Containing Lungs

**Figure 6:**
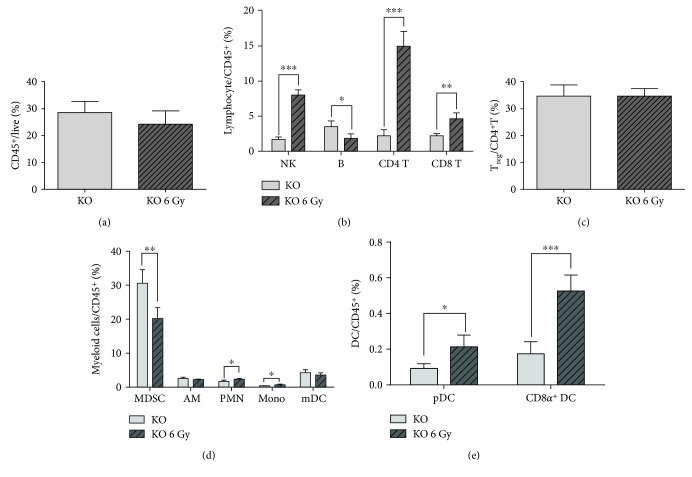
Irradiated *Oasl1* ^−*/*−^ mice contain higher cytotoxic effector immune cells in the tumor-containing lungs. WT and *Oasl1^−/−^* (KO) mice (*n* = 4 per group) were intravenously injected with TC-1 tumor cells (10^6^/mouse). At 7 d.p.i., the radiation-treatment groups of tumor-bearing WT and *Oasl1^−/−^* mice received 6 Gy whole body irradiation. At 21 d.p.i., the right inferior lobe of the lung was collected, and the lung-derived single cells were analyzed by FACS. (a) Summary of FACS data showing the percentage of CD45^+^ cells among live cells in the tumor-containing lung. (b) Summary of FACS data showing the percentage of NK cells (NK1.1^+^), B cells (CD19^+^), CD4 T cells (CD3^+^CD4^+^), and CD8 T cells (CD3^+^CD8^+^) among CD45^+^ cells. (c) Summary of FACS data showing the percentage of T_reg_ (CD4^+^Foxp3^+^) among CD4^+^ T cells in the lung. (d) Summary of FACS data showing the percentage of MDSC, AM, PMN, Mono, and mDC among CD45^+^ cells. (e) Summary of FACS data showing the percentage of pDC and CD8*α* ^+^ DC among CD45^+^ cells. ^∗^ *p* < 0.05, ^∗∗^ *p* < 0.01, and ^∗∗∗^ *p* < 0.001. Data are representative of at least three independent experiments.

To establish the cause for the enhanced resistance of irradiated *Oasl1* ^−*/*−^ mice, major immune cell composition in tumor-containing lungs was analyzed by FACS. At 21 d.p.i., the percentage of CD45^+^ hematopoietic cells in the lungs of irradiated *Oasl1* ^−*/*−^ mice was not significantly different from that of nonirradiated *Oasl1* ^−*/*−^ mice (Figure 6(a)). However, the proportion of major lymphocytes, such as CD4^+^ and CD8^+^ T cells and NK cells, within CD45^+^ hematopoietic cells was much higher (>2-fold) in the lungs of irradiated *Oasl1* ^−*/*−^ mice. Conversely, the proportion of B cells within CD45^+^ hematopoietic cells in the lungs of irradiated *Oasl1* ^−*/*−^ mice were lower (Figure 6(b)), which might be caused by a higher sensitivity of B cells to irradiation [34, 35]. Further, the proportion of T_reg_ within CD4^+^ T cells was not significantly changed in the lungs of irradiated *Oasl1* ^−*/*−^ mice (Figure 6(c)). At 21 d.p.i., the proportion of MDSCs was lower than that of untreated *Oasl1* ^−*/*−^ mice, while the proportion of AM within CD45^+^ cells in the lungs of irradiated *Oasl1* ^−*/*−^ mice were not significantly different from that of untreated *Oasl1* ^−*/*−^ mice (Figure 6(d)). However, the proportions of PMN and monocytes within CD45^+^ cells in the lungs of irradiated *Oasl1* ^−*/*−^ mice were slightly higher than those of untreated *Oasl1* ^−*/*−^ mice (Figure 6(d)). Among major dendritic cell populations, the proportion of mDC within CD45^+^ cells in the lungs of cisplatin-treated *Oasl1* ^−*/*−^ mice was not significantly different from that of untreated *Oasl1* ^−*/*−^ mice (Figure 6(d)). However, the proportions of pDC and CD8*α* ^+^ DC within CD45^+^ cells in the lungs of irradiated *Oasl1* ^−*/*−^ mice were higher than those of untreated *Oasl1* ^−*/*−^ mice (Figure 6(e)). Similar to cisplatin-treated *Oasl1* ^−*/*−^ mice, CD8^+^ T and NK cells (cytotoxic effector cells), as well as CD8*α* ^+^ DCs (major cells cross-presenting tumor antigen to CD8^+^ T cells), which were present in a much higher proportion within CD45^+^ cells in the lungs of irradiated *Oasl1* ^−*/*−^ mice, and MDSCs (present in a lower number in the lungs of irradiated *Oasl1* ^−*/*−^ mice) might contribute to the more effective antitumor immune response in the irradiated *Oasl1* ^−*/*−^ mice.

### 3.6. Radiation-Treated *Oasl1* ^−*/*−^ Mice Produce More IFN-*γ*, IFN-I, and Apoptotic Cells in the Tumor-Containing Lungs

**Figure 7:**
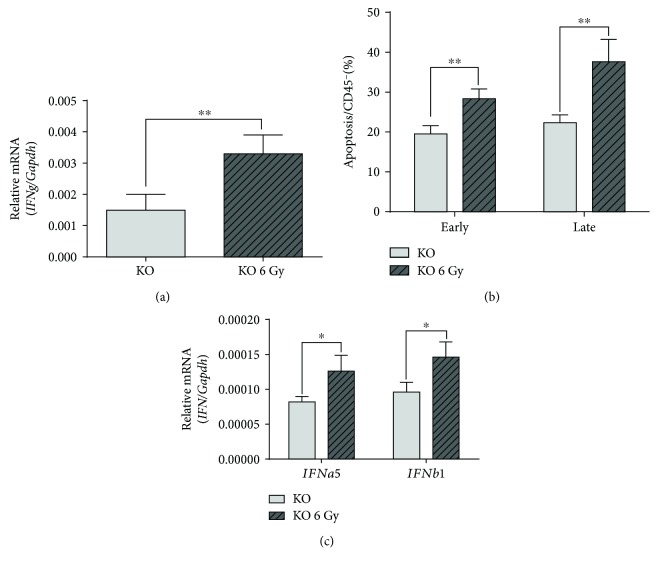
Irradiated *Oasl1* ^−*/*−^ mice produce more IFN-*γ,* IFN-I, and apoptotic cells in the tumor-containing lung. WT and *Oasl1^−/−^* (KO) mice (*n* = 4 per group) were intravenously injected with TC-1 tumor cells (10^6^/mouse). At 7 d.p.i., the radiation-treatment groups of tumor-bearing WT and *Oasl1^−/−^* mice received 6 Gy whole body irradiation. At 21 d.p.i., the right middle lobe of the lung was collected for RNA analysis and the right inferior lobe of the lung was collected for FACS analysis. (a–c) Quantitative RT-PCR analysis of mRNA expression for *IFNg* (a) and *IFNa5* and *IFNb1* (c); mRNA expression level (*n* = 4 per group) normalized to *Gapdh* is shown as relative mRNA. (b) Summary data (*n* = 4 per group) showing the percentage for two types of apoptotic cells among CD45^−^ cells; early apoptotic cells (Annexin V^+^/7-AAD^−^) and late apoptotic cells (Annexin V^+^/7-AAD^+^). ^∗^ *p* < 0.05, ^∗∗^ *p* < 0.01, and ^∗∗∗^ *p* < 0.001. Data are representative of at least three independent experiments.

To indirectly investigate the functionality of cytotoxic immune cells, IFN-*γ* expression was evaluated in the tumor-containing lungs at 21 d.p.i. by qRT-PCR. The expression level of IFN-*γ* mRNA was much higher in the lungs of irradiated *Oasl1* ^−*/*−^ mice compared to untreated *Oasl1* ^−*/*−^ mice (Figure 7(a)), indicating that cytotoxic cells were functionally more active in the lungs of irradiated *Oasl1* ^−*/*−^ mice. Consistently, the lungs of irradiated *Oasl1* ^−*/*−^ mice contained much more apoptotic cells within nonhematopoietic cells (CD45^−^ cells) than those of untreated *Oasl1* ^−*/*−^ mice (Figure 7(b)). These results indicate that irradiated *Oasl1* ^−*/*−^ mice have more tumor-attacking cytotoxic cells, such as CD8^+^ T cells and NK cells, that can kill growing tumors in the lungs, leading to the improved survival of irradiated *Oasl1* ^−*/*−^ mice.

We next investigated whether the enhanced antitumor immune response observed in the lungs of irradiated *Oasl1* ^−*/*−^ mice may be caused by increased IFN-I expression, similar to cisplatin-treated *Oasl1* ^−*/*−^ mice. Indeed, the mRNA expression levels of IFN-Is, such as *IFNa5* and *IFNb1*, in the lungs of irradiated *Oasl1* ^−*/*−^ mice at 21 d.p.i., were higher (approximately 1.4-fold) than those of untreated *Oasl1* ^−*/*−^ mice (Figure 7(c)).

## 4. Discussion

In this study, we evaluated whether the resistance of *Oasl1* ^−/−^ mice to implanted tumors can be further improved with conventional cancer therapies such as chemotherapy and radiotherapy. We found that *Oasl1* ^−/−^ mice showed more resistance to implanted tumors when treated with a representative chemotherapeutic agent cisplatin, as well as irradiation, than untreated *Oasl1* ^−/−^ mice. Additionally, we found that antitumor cytotoxic effector cells, such as CD8^+^ T cells and NK cells, as well as CD8*α* ^+^ DCs (major antigen cross-presenting cells), were more abundant in the tumor-containing lungs of cisplatin-treated and irradiated *Oasl1* ^−/−^ mice than untreated *Oasl1* ^−/−^ mice. However, the proportions of immunosuppressive MDSCs within the hematopoietic cells and T_reg_ within CD4^+^ T cells in the therapy-applied *Oasl1* ^−/−^ lungs were lower or similar, respectively, to those of untreated *Oasl1* ^−/−^ mice. Consistently, the mRNA expression of cytotoxic effector cytokine IFN-*γ*, produced by functionally active CD8^+^ T and NK cells, was higher in the therapy-applied *Oasl1* ^−/−^ lungs, and more apoptotic nonhematopoietic cells (that are thought to be mainly tumor cells) [28, 29] were observed in the therapy-applied lungs of *Oasl1* ^−/−^ mice compared to untreated *Oasl1* ^−/−^ mice.

In our previous study, we demonstrated that *Oasl1* ^−/−^ mice produced higher levels of IFN-I and were more resistant to the TC-1 tumor challenge than WT mice [22]. In our current study, we showed that cisplatin-treated or irradiated *Oasl1* ^−/−^ mice expressed higher levels of IFN-I in the tumor-containing lungs than untreated *Oasl1* ^−/−^ mice, indicating that there is synergy between OASL1 deficiency and cisplatin treatment or irradiation for IFN-1 expression. Cisplatin and irradiation can directly kill growing tumor cells [36, 37], thus more tumor antigen and danger-associated molecular patterns (DAMPs), including tumor cell DNAs, ATP, and HMGB1, would be released from dying tumor cells in the therapy-applied *Oasl1* ^−/−^ lungs [36–38]. Tumor cell-derived DNAs can be detected by one of the major DNA sensors, cyclic GMP-AMP synthase (cGAS) (a cytosolic DNA sensor), which activates STING and then the TBK1 (the major kinase for IRF7 and IRF3 activation) pathway, thus inducing IFN-1 expression [39, 40]. According to our previous study using the same tumor model, pDC, which can predominantly produce large amounts of IFN-I because of its high basal expression of IRF7 [41, 42], was the main producer of IFN-I in tumors [22]. Thus, the cGAS-STING-TBK1-IRF7 pathway in pDC might also be responsible for IFN-I expression in the cisplatin-treated and irradiated tumor-containing lungs of *Oasl1* ^−/−^ mice, although we cannot rule out the possibility of the contribution of the TLR9-Myd88-IRF7 pathway, which can detect extracellular DNA in pDC [43, 44]. Regardless of the mechanism to detect the tumor cell DNA, the higher number of pDCs present in cisplatin-treated *Oasl1* ^−/−^ lungs (a greater number of CD45^+^ cells present in the lungs and a similar percentage of pDC within CD45^+^ cells lead to a higher number of pDCs in the lungs) and irradiation-treated *Oasl1* ^−/−^ lungs (similar number of CD45^+^ cells present in lungs and higher percentage of pDC within CD45^+^ cells lead to higher number of pDCs in the lungs) compared to untreated *Oasl1* ^−/−^ lungs, together with the higher availability of tumor cell-derived DNA for the pDC activation, would considerably contribute to the observed enhanced IFN-I expression in the therapy-applied *Oasl1* ^−/−^ lungs.

IFN-I is a potent immunostimulator that enhances host antitumor immune responses [10, 45]. IFN-I can promote the tumor antigen cross-presentation to naïve CD8^+^ T cells by stimulating DC (including CD8*α* ^+^ DCs) maturation and migration to lymph nodes and thus clonal expansion and differentiation into cytotoxic effector CD8^+^ T cells indirectly. IFN-I can also directly enhance clonal expansion of antigen-experienced CD8^+^ T cells and enhance CD8^+^ T cell cytotoxicity [46, 47]. Additionally, IFN-I can directly promote CD4^+^ T cell expansion and Th1 (type 1 T helper) cell differentiation [45, 48] and indirectly promote the expansion and survival of NK cells through the induction of IL-15 expression [49, 50]. Furthermore, IFN-I can promote B cell survival, differentiation, and function and inhibit the immunosuppressive function of T_reg_ [10, 51]. Collectively, these facts indicate that the higher amount of IFN-I present in the therapy-applied *Oasl1* ^−/−^ lungs would stimulate both innate and adaptive immune cells and provide a higher number and better functioning of cytotoxic effector cells to the therapy-applied *Oasl1* ^−/−^ lungs, leading to improved tumor resistance and better survival of the treated *Oasl1* ^−/−^ mice.

We used cisplatin as a main anticancer chemotherapeutic agent in this study. Cisplatin, a widely used chemotherapeutic agent in clinical practice, can cross-link DNA and directly induce apoptotic tumor cell death [25]. In addition to the direct cytotoxic activity, cisplatin can modulate the immune system to enhance the antitumor response [25, 36]. It can induce MHC I upregulation on the tumor and antigen-presenting cells. Further, it promotes proliferation and recruitment of immune effector cells, such as CD8^+^ T and NK cells. Finally, it enhances the lytic activity of cytotoxic effector cells and reduces the number of MDSC cells. Thus, the synergistic antitumor effect observed between OASL1 deficiency and cisplatin is thought to be caused by the combination of IFN-I's immune-boosting effects as well as cisplatin's direct cytotoxicity and indirect immune-boosting functions. Other chemotherapeutic agents showing immune-boosting roles in addition to the tumor cell cytotoxicity are worth exploring for potential synergy with OASL1 deficiency in the future [52, 53].

We also tested whether another major conventional cancer therapy, radiotherapy, can add an anticancer therapeutic benefit with OASL1 deficiency. Similar to cisplatin treatment, radiation increased the anticancer therapeutic benefits of OASL1 deficiency. This result indicates that OASL1 inhibition could be well integrated with conventional anticancer therapies as a combinatorial treatment. Although we did not explore the possibility of the potential synergy between OASL1 deficiency and targeted therapy or other immunotherapy in this study, OASL1 deficiency may show a good synergy with at least some of such therapies because IFN-I delivery has been shown to add a benefit to the targeted therapies using EGFR-targeting Abs (erlotinib and nimotuzumab) [54] and BRAF inhibitor [55] and immunotherapies using anti-PD-1 and anti-CTLA-4 Abs [56].

OASL1 is a translation inhibitor of IRF7, the IFN-inducible IFN-I master TF. Thus, it negatively regulates robust IFN-I production upon virus and tumor challenge [19–22]. Since there are other negative regulators acting on the process of IFN-I production and/or IFN-I receptor signaling pathway [17, 18], inhibitors of these other negative regulators might be useful together with conventional anticancer therapies to achieve a synergistic antitumor effect. Thus, developing specific inhibitors for such negative regulators, including OASL1, is worthy of investigation in the future.

## 5. Conclusions

In this study, we investigated whether suppression of OASL1 showed a synergistic effect on tumor clearance with the conventional cancer therapies, chemotherapy and radiotherapy, using *Oasl1* ^−*/*−^ mice and a lung metastatic tumor cell model. We found that *Oasl1* ^−*/*−^ mice treated with the anticancer drug cisplatin and irradiated *Oasl1* ^−*/*−^ mice showed enhanced resistance to injected tumors compared to untreated *Oasl1* ^−*/*−^ mice. We also found that the therapy-applied *Oasl1* ^−*/*−^ mice contained more cytotoxic effector cells, such as CD8^+^ T cells and NK cells, and produced more cytotoxic effector cytokine IFN-*γ* as well as IFN-I in their tumor-containing lungs compared to untreated *Oasl1* ^−*/*−^ mice. Collectively, these results show that OASL1-targeted therapy together with conventional cancer therapies could enhance tumor treatment efficacy.

## Data Availability

The data used to support the findings of this study are available from the corresponding author upon request.

## Supplementary Materials

Supplementary Materials Supplementary Figure 1: gating strategy used to identify major lymphocyte populations of NK, B, CD3^+^ T, CD4^+^ T, CD8^+^ T, and T_reg_ cells. Supplementary Figure 2: gating strategy used to identify major pulmonary myeloid populations of PMN, monocytes (Mono), AM, mDC, and MDSC from tumor-containing mouse lung tissues.
