# Supplementary material for: Enhanced Antitumor Immune Response in 2′-5′ Oligoadenylate Synthetase-Like 1- (OASL1-) Deficient Mice upon Cisplatin Chemotherapy and Radiotherapy
Source: J Immunol Res. 2019 Mar 31;2019:7596786. doi: 10.1155/2019/7596786 (PMC6462330; doi:10.1155/2019/7596786)
Supplement: Supplementary Materials — Supplementary Figure 1: gating strategy used to identify major lymphocyte populations of NK, B, CD3+ T, CD4+ T, CD8+ T, and Treg cells. Supplementary Figure 2: gating strategy used to identify major pulmonary myeloid populations of PMN, monocytes (Mono), AM, mDC, and MDSC from tumor-containing mouse lung tissues. [file 7596786.f1.pdf]

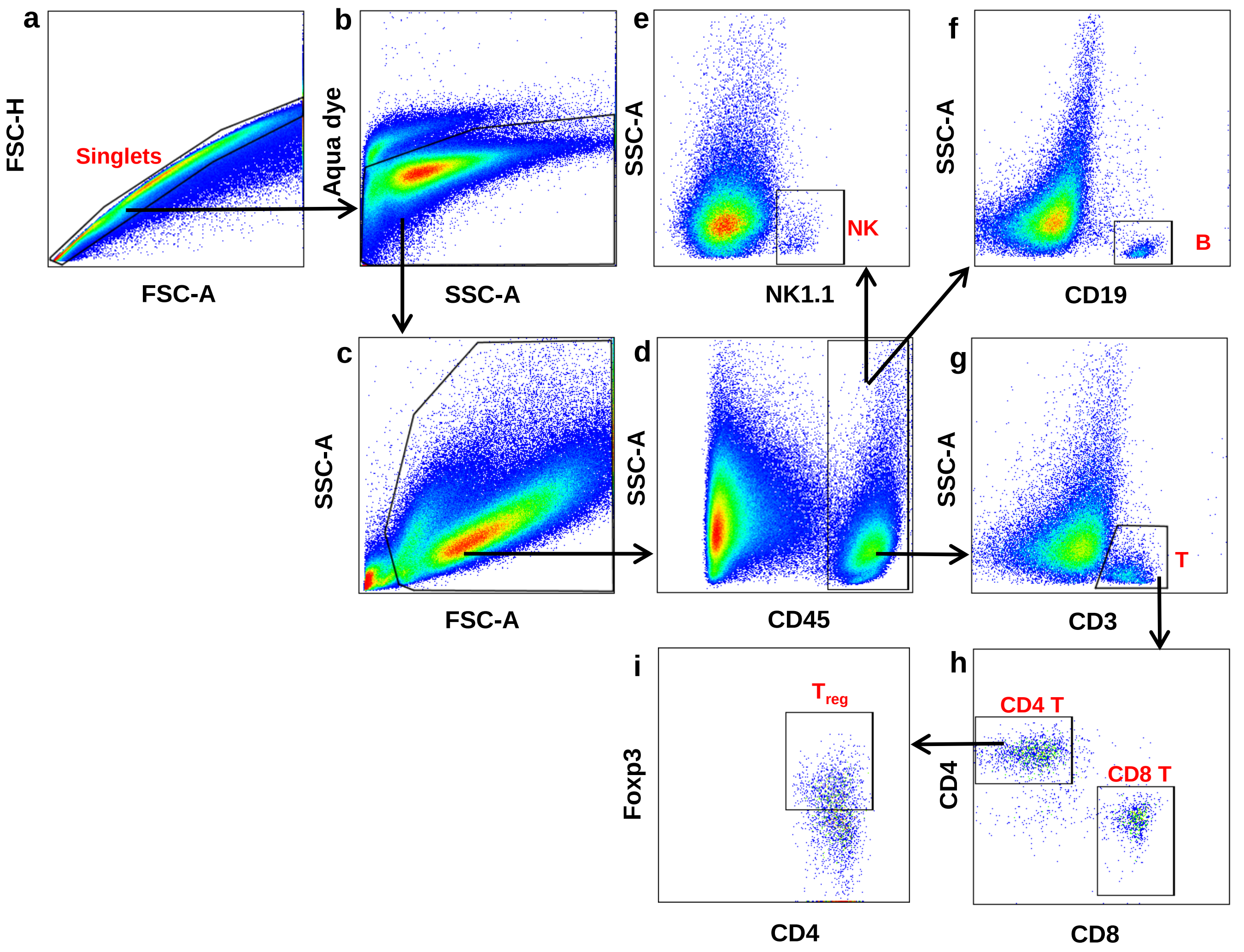

Supplementary Figure 1: Gating strategy used to identify major lymphocyte populations, NK, B, $CD3^+$ T, $CD4^+$ T, $CD8^+$ T, and T_{reg} cells. Tumor-containing lung-derived single cells were stained with Aqua fluorescent reactive dye (dead cell staining) together with either fluorophore-labeled anti-CD45, CD3, CD4, CD8, CD19, and NK1.1 antibodies (Abs) or fluorophore-labeled anti-CD45, CD3, CD4, CD8, CD19, and Foxp3 Abs to identify various cell subsets. (a) Doublets deviating from the diagonal in the plot of forward scatter area (FSC-A) versus forward scatter height (FSC-H) were excluded. (b) Following, dead cells were excluded by their staining with Aqua fluorescent reactive dye. (c) Live cells, which were higher up on the FSC-A and side scatter area (SSC-A) profile, were further selected. (d) $CD45^+$ hematopoietic cells were then selected. (e) Subsequently, NK cells ($NK1.1^+SSC\text{-}A^{low}$) and (f) B cells ($CD19^+SSC\text{-}A^{low}$) were identified. (g) T cells ($CD3^+SSC^{low}$) were identified and gated. (h) $CD4^+$ T cells ($CD3^+CD4^+CD8^-$) and $CD8^+$ T cells ($CD3^+CD4^-CD8^+$) were identified and (i) T_{reg} ($CD3^+CD4^+CD8^-Foxp3^+$) were identified among $CD4^+$ T cells. Isotype-matched controls for all Abs were used to determine positive populations. Identified populations are marked in red text.

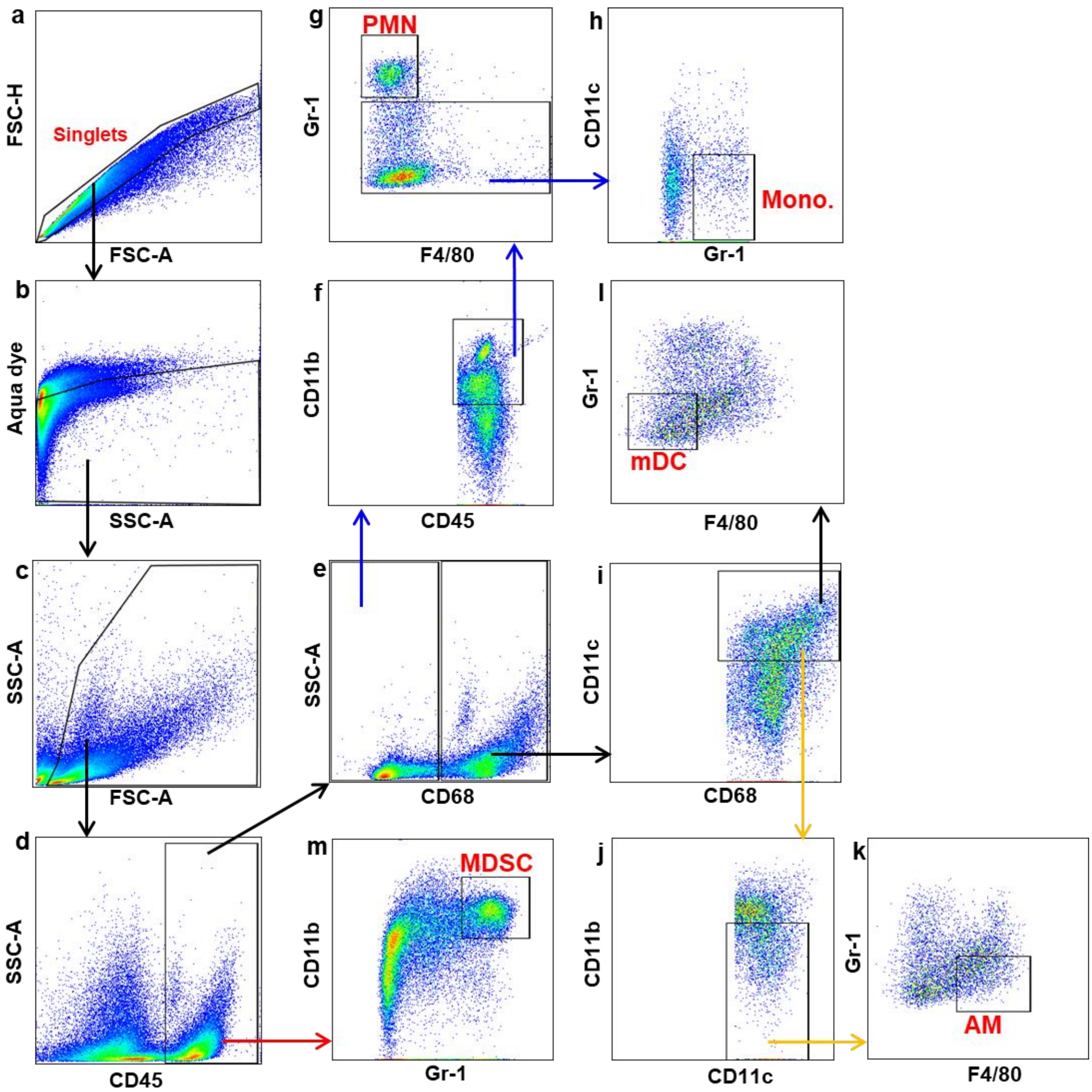

Supplementary Figure 2: Gating strategy used to identify major pulmonary myeloid populations PMN, Monocyte (Mono), AM, mDC, and MDSC from tumor-containing mouse lung tissues. This strategy is based on the report by Rinat Zaynagetdinov group at Vanderbilt University (26). Lung-derived single cells were stained with fluorophore-labeled anti-CD45, CD68, CD11b, F4/80, CD11c, and Gr-1 Abs together with Aqua fluorescent reactive dye (dead cell staining). (a) Doublets deviating from the diagonal in the plot of FSC-A versus FSC-H were excluded. (b) Viable Aqua dye-negative cells were then selected, and (c) live cells with higher FSC/SSC profile were further selected. (d) $CD45^+$ cells were selected, and (e) $CD68^{hi}$ and $CD68^{low}$ cells were separately gated. (f) $CD68^{low}CD11b^+$ cells were selected. (g) PMN (Polymorphonuclear cell, $CD68^{low}CD11b^+F4/80^-Gr\text{-}1^{hi}$) was identified, and $CD68^{low}CD11b^+Gr\text{-}1^{low}$ cells were gated. (h) Monocyte (Mono, $CD68^{low}CD11b^+Gr\text{-}1^{low}CD11c^-$) were identified. (i) $CD68^{hi}CD11c^+$ cells were gated, and (j) $CD68^{hi}CD11c^+CD11b^-$ cells were selected. (k) AM (Alveolar macrophage, $CD68^{hi}CD11c^+CD11b^-F4/80^+Gr\text{-}1^-$) were identified. (l) mDC ($CD68^{hi}CD11c^+F4/80^-Gr\text{-}1^-$) were identified, and (m) broadly defined MDSC cells ($CD11b^+Gr\text{-}1^+$) were identified. Isotype-matched controls for all Abs were used to determine positive populations. Identified populations are marked in red text.
